# Reduced striatal dopamine transmission as a transdiagnostic substrate of psychomotor retardation

**DOI:** 10.1093/brain/awaf335

**Published:** 2025-09-12

**Authors:** Ian Lam Leong, Tsz Huen Ng, Kunal Sen, Ella Burchill, Harry Costello, James B Badenoch, Jan Coebergh, Robert A McCutcheon, Akshay Nair, Michael Browning, Quentin J M Huys, Glyn Lewis, Andrew Lees, Anthony S David, Jonathan P Rogers

**Affiliations:** Division of Psychology and Language Sciences, University College London, London WC1H 0AP, UK; Faculty of Education, University of Hong Kong, 999077 Hong Kong; Faculty of Life Sciences, University College London, London WC1E 6BT, UK; Division of Psychiatry, University College London, London WC1H 0AL, UK; Division of Psychiatry, University College London, London WC1H 0AL, UK; Department of Psychological Medicine, Institute of Psychiatry, Psychology and Neuroscience, King's College London, London SE5 8AF, UK; South London and Maudsley NHS Foundation Trust, London SE5 8AZ, UK; Ashford St Peter’s Hospital and St George’s Hospitals, Ashford TW15 3AA, UK; Department of Psychiatry, University of Oxford, Oxford OX3 7JX, UK; Oxford Health NHS Foundation Trust, Oxford OX3 7JH, UK; Department of Psychosis Studies, Institute of Psychiatry, Psychology & Neuroscience, King’s College London, London SE5 8AF, UK; Department of Neurology, St. George’s University Hospitals NHS Foundation Trust, London SW17 0QT, UK; Department of Neurology, City St George’s University of London, London SW17 0RE, UK; Department of Psychiatry, University of Oxford, Oxford OX3 7JX, UK; Division of Psychiatry, University College London, London WC1H 0AL, UK; Max Planck UCL Centre for Computational Psychiatry and Ageing Research, Department of Imaging Neuroscience, Queen Square Institute of Neurology, University College London, London WC1N 3BG, UK; Division of Psychiatry, University College London, London WC1H 0AL, UK; Reta Lila Weston Institute of Neurological Studies, Queen Square Institute of Neurology, University College London, London WC1N 3BG, UK; Division of Psychiatry, University College London, London WC1H 0AL, UK; Division of Psychiatry, University College London, London WC1H 0AL, UK; Department of Neuropsychiatry, National Hospital for Neurology and Neurosurgery, London WC1N 3BG, UK

**Keywords:** basal ganglia, striatum, movement disorders, bradykinesia, cognitive slowing

## Abstract

Psychomotor retardation, defined as generalized slowing of movement and speech, is a feature of several neurological and psychiatric disorders. In this review, we discuss the hypothesis that reduced striatal dopaminergic transmission is a transdiagnostic substrate for psychomotor retardation underlying the motor features of conditions such as Parkinson’s disease, drug-induced parkinsonism, neuroleptic malignant syndrome, catatonia and depression. We examine the evidence across clinical, epidemiological, neuroimaging, laboratory and therapeutic studies.

Parkinsonian disorders share slowed movement and a reduction in verbal output with catatonia and depression. Bradyphrenia, slowed cognitive processing, also occurs in Parkinson’s disease and depression. In addition, there are close epidemiological relationships between depression and Parkinson’s disease, and between catatonia and neuroleptic malignant syndrome.

Neuroimaging studies also generally support the association of psychomotor retardation with reduced dopaminergic transmission, particularly in the dorsal striatum. CSF measurement of homovanillic acid (a dopamine catabolite) yields inconsistent results and is non-specific. Parkinson’s disease and catatonia generally respond well to dopaminergic medication. In contrast, dopamine antagonists can induce both parkinsonism and catatonia.

Our review is limited by the variability in measurement of psychomotor retardation and difficulty distinguishing between cognitive and motor slowing. It is also likely that other neurotransmitters, such as GABA and serotonin, play an important role in psychomotor speed.

It is possible that dopaminergic deficits in psychiatric disorders represent functional disruptions, in contrast to the structural damage to the substantia nigra in Parkinson’s disease. We propose further research be conducted into the effects of levodopa and dopamine agonists in depression with psychomotor retardation. Alternative neuroimaging methods such as PET sequences with shorter imaging protocols and neuromelanin-MRI should also be explored.

## Teaser

Leong *et al.* propose that psychomotor retardation in disorders such as Parkinson’s disease, catatonia, and depression may share a common mechanism—reduced striatal dopaminergic function—supported by neuroimaging findings, clinical overlap, and treatment responses.

## Introduction

Psychomotor retardation (PMR) is a generalized slowing of movement and speech.^1,2^ The concept describes difficulties in initiation and maintenance of movements as well as differences between internally and externally generated thoughts or movements.^3-5^ Assessment of PMR takes into account an individual’s age, culture, baseline activity and environmental factors such as temperature.

Patients with a range of psychiatric and neurological conditions present with PMR, but the term has been particularly associated with depression, especially melancholic depression.^6,7^ Given that as few as 27% of patients with depression experience remission with untargeted first-line treatment,^8^ there is an urgent need to identify why certain individuals respond to particular treatments. Uncovering transdiagnostic mechanisms for PMR could provide clinicians with novel therapies for depression and other conditions featuring PMR by repurposing medications effective in PMR for other disorders.

**Figure 1:**
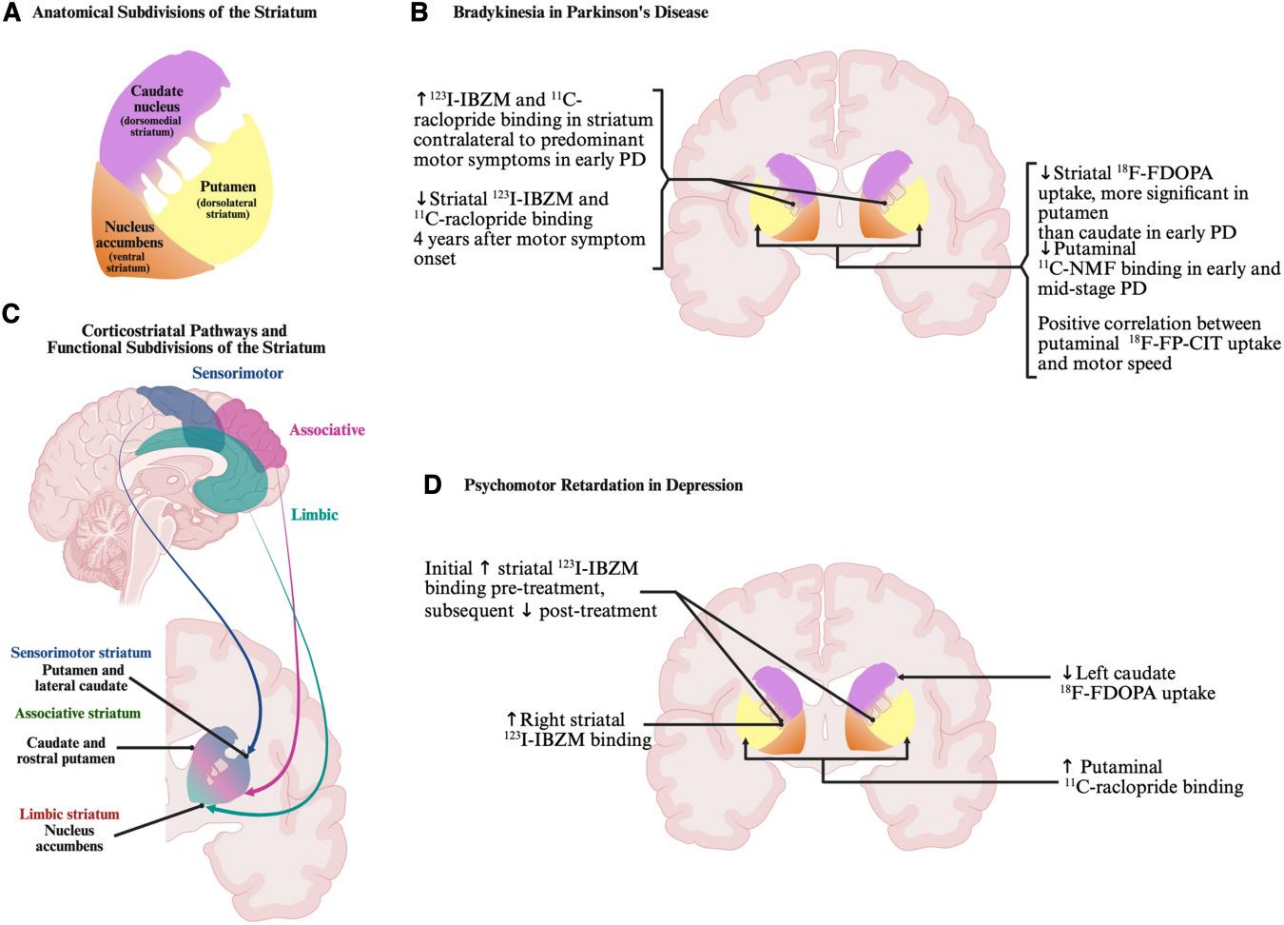
**Anatomy and functional architecture of the striatum with relevant dopaminergic imaging findings in Parkinson’s disease and depression**. (**A**) Anatomical subdivisions of the striatum. The striatum is anatomically divided into the nucleus accumbens, caudate nucleus and putamen, the latter two being separated by the internal capsule. (**B**) Neuroimaging findings corresponding to bradykinesia in Parkinson’s disease. A range of neuroimaging modalities implicate the striatum in the bradykinesia of Parkinson’s disease and—where there is greater anatomical precision—studies have tended to emphasize the role of the putamen. (**C**) Corticostriatal pathways and functional subdivisions of the striatum. Most of the cortex projects to the striatum, and there is some anatomical overlap in function, but in broad terms, there is a dorsal-to-ventral gradient from sensorimotor striatum to associative striatum to limbic striatum. (**D**) Neuroimaging findings corresponding to psychomotor retardation in depression. Fewer studies have examined psychomotor retardation in depression, but there is converging evidence implicating the striatum, though less is known about the anatomical specificity within the striatum. ^11^C-NMF = ^11^C-nomifensine; ^123^I-IBZM = iodine-123-iodobenzamide; ^123^I-FP-CIT = *N*-ω-fluoropropyl-2β-carbomethoxy-3β-(4-iodophenyl)tropane/^123^I-ioflupane; ^18^F-FDOPA = 6-^18^F-fluoro-L-3,4-dihydroxyphenylalanine; PD = Parkinsons' disease. Created in BioRender. Leong I. (2025) https://BioRender.com/swnk7a0.

Central to the neuroanatomy of voluntary movement are thought to be the cortico-striato-thalamocortical circuits, particularly those involving the dorsal striatum, although the distinction from the ventral striatum is not entirely clear either in terms of cytoarchitecture or histochemistry.^9^ Structural and functional imaging, however, attest to a gradient from medial to lateral and from rostral to caudal in terms of projections from the cortex,^10^ which corresponds to a ventral striatum that serves limbic areas of cortex, as opposed to a dorsal striatum serving sensorimotor and associative areas.^9^ The dorsal striatum is in turn anatomically segregated by the internal capsule into the putamen and caudate (Fig. 1A). Projections from the sensorimotor cortex converge on the dorsolateral putamen and the lateral caudate nucleus, while association cortex projects to the head of the caudate and the more ventromedial putamen (Fig. 1C).^11,12^

The mechanisms involved in programming voluntary movement are considered to involve the balance of the direct and indirect pathways of the basal ganglia. In the direct pathway, glutamate is released at the striatum, activating inhibitory GABAergic medium spiny neurons projecting to the internal globus pallidus and substantia nigra pars reticulata.^13,14^ This causes disinhibition of thalamic glutamatergic neurons that project back to the cortex and of brainstem motor regions (specifically the midbrain locomotor region and the pedunculopontine tegmental nucleus), which are critical to posture and locomotion.^13-16^ In the indirect pathway, striatal GABAergic activity reduces the GABA output of the external globus pallidus, disinhibiting the glutamatergic neurons of the subthalamic nucleus, which excite the internal globus pallidus and substantia nigra pars reticulata.^13,14^ This increases inhibition of the thalamic neurons projecting to the cortex, reducing motion.^13-15^ Dopamine has a crucial modulatory role in both the direct and indirect pathways. Dopamine D_1_ receptors (D_1_Rs) are expressed at higher levels on direct pathway striatal neurons and have an excitatory effect, whereas D_2_ receptors (D_2_Rs) have greater expression on indirect pathway striatal neurons and have an inhibitory effect.^15^ A reduction in striatal dopaminergic transmission would thus increase the activity of the indirect pathway whilst decreasing activity in the direct pathway, having a dual effect of inhibiting the initiation and speed of movement. The bradykinesia of Parkinson’s disease is therefore—in simple terms—conceived as overactivity of the indirect pathway relative to the direct pathway, although more complex models are being developed.^17^

Reduced striatal dopaminergic transmission is present in Parkinson’s disease due to loss of dopaminergic neurons in the substantia nigra pars compacta.^18^ However, a similar functional disturbance of striatal dopamine signalling has separately been proposed to be present in catatonia,^19^ neuroleptic malignant syndrome,^20^ drug-induced parkinsonism^21^ and in the pathophysiology of depression with PMR.^22^ In the cognitive domain, Daniel Rogers *et al*.^23^ proposed that bradyphrenia (slowed cognitive processing) in Parkinson’s disease and PMR in depression both involve dopaminergic dysfunction, based on a study using a computerized test showed similarities between the two. Lately, Hinkle and Pontone^24^ have also suggested that catatonia be considered ‘psychiatric parkinsonism’, based on recent neuroimaging evidence.

In this paper, we present the hypothesis that reduced dopaminergic transmission in the striatum (particularly the dorsal striatum) is a transdiagnostic mechanism underlying PMR, using evidence from clinical features, epidemiology, laboratory studies and neuroimaging, as well as response to dopaminergic treatments. Integrating findings from these research modalities and relating them to developments in cognitive neuroscience, we discuss how the theory applies to conditions as diverse as Parkinson’s disease, drug-induced parkinsonism, neuroleptic malignant syndrome, catatonia, depression and obsessional slowness.

## Clinical presentation

Overlapping terminology is used to describe slowed movement and cognition in neurology and psychiatry. We present some of the commonly used terms in Table 1.

**Table 1:** Terminology used for concepts related to psychomotor retardation in neurology and psychiatry

Term	Selected conditions in which it is commonly described	Definition
Psychomotor retardation	Depression, schizophrenia^5,20^	Generalized slowing of movement and speech^2^
Bradyphrenia	Parkinson’s disease, Huntington’s disease^21^	Slowing of thought^21^
Bradykinesia	Parkinson’s disease, other parkinsonisms^22^	Slowed movement with a progressive reduction in amplitude or speed^22^
Hypokinesia	Parkinson’s disease, Huntington’s disease, other parkinsonisms^23,24^	A reduction in the quantity of movement^24^
Catatonia	Depression, schizophrenia, NMDA receptor encephalitis^25,26^	A syndrome involving signs of decreased, increased or abnormal psychomotor activity, including stupor, mutism and posturing^2^
Akinesia	Akinetic mutism^24,a^	Absence of movement^24^

NMDA = *N-*methyl-D-aspartate.

^a^Akinesia has sometimes been given a more liberal definition as a synonym for bradykinesia.^24^

Before addressing the issue of a transdiagnostic substrate for PMR, we will examine the similarities (and differences) in clinical presentation between Parkinson’s disease, where the role of dopamine is established, and other conditions where it is more speculative. Reduced dopaminergic transmission in the dorsal striatum leads to some of the cardinal motor signs of Parkinson’s disease, specifically bradykinesia (slowness, with progressive decrement in amplitude and speed of self-initiated movement) and rigidity,^25^ although its role in tremor is probably more complex.^26^ Speech in Parkinson’s in some ways parallels the bradykinesia, in that there is a delayed onset,^27,28^ but rapid and hesitating speech may also occur,^29^ mirroring the festinant parkinsonian gait, consisting of a progressive shortening of step length.^30^

Bradykinesia is not specific to Parkinson’s disease, but it is quite specific to reduced striatal dopamine transmission. It is a feature of related parkinsonian syndromes, such as multiple system atrophy, progressive supranuclear palsy and corticobasal degeneration all of which have severe neuronal loss in the pars compacta of the substantia nigra, although it is not uniform and patients with supranuclear palsy seem to lack the decrement present in Parkinson’s disease.^31,32^ Drug-induced parkinsonism—usually due to antagonists of the dopamine D_2_R—also features bradykinesia and rigidity as important diagnostic signs.^33^ Acutely, antagonists at the D_2_R can occasionally trigger neuroleptic malignant syndrome, which consists of the more extreme akinesia, alongside rigidity, altered consciousness, hyperpyrexia and autonomic disturbance.^34,35^ Bradykinesia sometimes also occurs in hyperkinetic disorders, such as dystonia, chorea and Huntington’s disease,^36^ although it should be noted that Huntington’s disease has a different dopaminergic mechanism driven by the selective degeneration of striatal medium spiny neurons.^37^

Psychomotor speed in depression is reduced, with meta-analytic evidence finding a standardized mean difference in actigraphically measured psychomotor speed of −0.78 (95% CI: −0.99 to −0.57) compared to healthy controls.^38^ Between 25% and 50% of patients with depression exhibit visible PMR,^39^ and it is strongly correlated with depression severity.^40^ The PMR of depression can present very similarly to the bradykinesia of Parkinson’s disease, with comparable presentations of a slow gait, hunched posture, expressionless facies, alongside slow movement and quiet speech, although usually to a lesser degree.^41-43^ The performance of patients with depression in certain cognitive tasks appears to be related to their motor slowing.^23^ Perhaps an even closer comparison may be made with catatonia, a neuropsychiatric syndrome of various aetiologies, classically consisting of stupor, mutism and behavioural withdrawal.^44^ A comparison of clinical features between Parkinson’s disease and catatonia is shown in Table 2, illustrating similarities in muscle tone, speed and quantity of movement, facial expression, freezing episodes and altered mental status. There is an entity of excited catatonia, which may alternate with stuporous states,^44,53^ suggesting that either catatonia is not a pathophysiologically homogeneous state, or that there are rapid fluctuations in the neurochemistry of the basal ganglia. Importantly, the onset of catatonia tends to be subacute, unlike the gradual onset seen in Parkinson’s disease.

**Table 2:** Comparison of shared clinical features between Parkinson’s disease and catatonia

	Parkinson’s disease^45-48^	Catatonia^47,49-52^
**Motor**		
Rigidity	Present, especially cogwheel rigidity, increasing with activation	Often present, but tone may also be normal or reduced. Catalepsy is highly specific.
Hypokinesia (slowed or reduced movement)	Bradykinesia, akinesia	Stupor (decreased psychomotor movement and reactivity), akinesia
Tremor	Present at rest and postural	Not typically observed
Facial manifestations	Masked facies, reduced spontaneous blink rate	Flat affect, staring, reduced blink rate
Sudden motor freezing	Freezing of gait (sudden, unwanted inability to move) typically triggered by external stimuli	Motor blocking (*Sperrung;* abrupt cessation in voluntary movement or speech), may not have external trigger
**Non-motor**		
Altered mental status	Depression, apathy, anhedonia, anxiety, psychosis, impulse control disorders	Depression, mania, anxiety, psychosis

We have shown that some neurological and psychiatric conditions share PMR. Motor slowing also appears to be closely related to mental processes at the level of analysis of the individual condition. In Parkinson’s disease, bradykinesia is strongly correlated with bradyphrenia—slowed cognitive processing.^54^ There is, moreover, a correlation between bradykinesia and some of the affective dimensions of Parkinson’s disease, specifically anhedonia and apathy.^45-49,55^ Anhedonia and apathy are both thought to be related to the dopaminergic deficits, although the specific localization is possibly more likely to be in mesocorticolimbic pathways, rather than the nigrostriatal projections thought to be responsible for bradykinesia.^46,50-52^ Cognition is also likely to be relevant to these affective states, as executive dysfunction is closely related to apathy.^56,57^ However, while bradykinesia is correlated with anhedonia and apathy, these are phenomenologically distinct concepts and do not always coexist.^58,59^

In psychiatric conditions as well, there is evidence that PMR is closely linked to the affective state. In obsessional slowness, a severe form of obsessive-compulsive disorder, there is difficulty initiating voluntary action and often severely slowed movement.^60^ In depression, PMR correlates with anhedonia,^61^ mirroring the association seen with bradykinesia in Parkinson’s disease.

In summary, the bradykinesia seen in Parkinson’s disease and other neurological movement disorders shows phenomenological similarities to the PMR of various psychiatric conditions, although subtler aspects such as the motor decrement are not clearly present in psychiatric conditions. There is also a relationship between PMR and states such as anhedonia and apathy, which are thought to be related to reduced dopamine transmission.

## Epidemiology

There is epidemiological evidence to suggest that depression is related to the development of parkinsonian disorders. Two meta-analyses have found an increased risk of subsequent Parkinson’s disease in patients with depression, with an odds ratio of 2.04 (95% CI: 1.02–4.08) and relative risk of 2.20 (95% CI: 1.87–2.58), respectively.^62,63^ Intervals of between 2 and 16 years between onset of depression and Parkinson’s disease diagnosis have been reported.^62^ The results of a recent Mendelian randomization study suggest that depression has a causal role in the development of Parkinson’s disease.^64^ The largest genome-wide association study of depression to date found that one of the genes with the strongest evidence was the dopamine receptor D_2_ (*DRD2*) gene.^65^ There is some evidence that PMR may constitute a genetically distinct form of depression,^66^ but we are not aware of studies that have investigated whether this group is particularly at-risk for developing Parkinson’s disease.

As a rarer disorder, evidence for catatonia is weaker. Catatonia has occasionally been reported to co-occur with Parkinson’s disease, dementia with Lewy bodies and progressive supranuclear palsy.^67-76^ In some cases, catatonia has been reported in the context of Parkinson’s disease after withdrawal of pro-dopaminergic medications.^72,76^ Parkinsonism and catatonia have also been noted to co-occur in certain genetic syndromes, including in Rett syndrome^77^ and in a patient with variants of the *SHANK3* and *SYNJ1* genes.^78^ These reports are suggestive but by no means conclusive.

Catatonia is, however, convincingly linked to the development of neuroleptic malignant syndrome. Catatonia may co-exist with neuroleptic malignant syndrome, and some authors have suggested that neuroleptic malignant syndrome is merely a subtype of malignant catatonia.^79^ Even if one does not accept this contention—and there are features such as diaphoresis, rigor, fever and tremor that are relatively specific to neuroleptic malignant syndrome^80^—the epidemiological relationship between the two conditions is compelling. Catatonia has been found to precede,^81^ co-exist with^82^ and follow neuroleptic malignant syndrome.^83-85^ Among 1719 patients with schizophrenia, one study found that the odds ratio for catatonia was 87.0 (95% CI 25.3–462.8) if neuroleptic malignant syndrome was present.^86^ There also appears to be a spectrum between antipsychotic-induced catatonia and neuroleptic malignant syndrome.^87^

In summary, depression is robustly linked to the subsequent development of Parkinson’s disease. Case reports and series suggest a relationship between catatonia and various parkinsonian disorders, but there is now convincing epidemiological evidence of a relationship to neuroleptic malignant syndrome.

## Laboratory evidence

Homovanillic acid (HVA) is the end-product of dopamine metabolism and can be measured in CSF as a biomarker of central dopaminergic activity.^88^ HVA is reduced in Parkinson’s disease^89-91^ and correlates with disease progression. A small study of neuroleptic malignant syndrome also found reduced HVA, which persisted beyond recovery, suggesting an underlying hypo-dopaminergic predisposition.^92^

Studies on whether HVA is reduced in individuals with depression compared to healthy controls have yielded inconsistent results,^93-96^ as have those comparing depressed patients with and without a melancholic subtype.^97,98^ Two studies have examined the relationship between PMR and HVA in patients with depression, finding no correlation.^99,100^ In one study, patients with depression and PMR had lower CSF HVA concentrations than healthy controls and those with depression without PMR, but the numbers were small and did not reach statistical significance.^99^ One study has found that HVA levels were positively correlated with psychomotor activity, but this included patients with depression and mania, so it is possible that the finding was driven by the latter group.^101^

In catatonia, one study found increased HVA levels relative to healthy controls.^102^ The same group found no relationship between an excited or retarded catatonia phenotype and HVA levels, although lorazepam responders appeared to have higher levels.^103^

Before trying to interpret these findings, it is worth providing some caveats about the measurement of CSF HVA. Such measurement is neuroanatomically non-specific; that is, it does not allow us to ascertain the extent of dopamine metabolism in a particular part of the brain. Second, it is temporally non-specific; that is, it is unable to capture rapid fluctuations in dopamine metabolism, such as those that may occur during phasic release associated with certain behavioural responses.^104^ This may be particularly relevant to psychiatric disorders, where there may be a functional dopaminergic deficit without the corresponding structural pathology in the substantia nigra that is present in Parkinson’s disease.

Evidence from CSF studies strongly supports the role of dopamine in Parkinson’s disease. Evidence for other conditions is much weaker, but the limitations of the method and the small studies conducted do not contradict our hypothesis. The studies do suggest that at some level the effect in depression and catatonia is different or at least more transient than in Parkinson’s disease. Northoff *et al*.^102^ hypothesized that the elevated HVA levels in patients with catatonia are due to increased mesolimbic dopaminergic transmission, which in turn downregulates nigrostriatal transmission, causing catatonia. Whilst this is largely speculative, it is compatible with the neuroimaging evidence we present later in this paper.

## Neuroimaging

**Figure 2:**
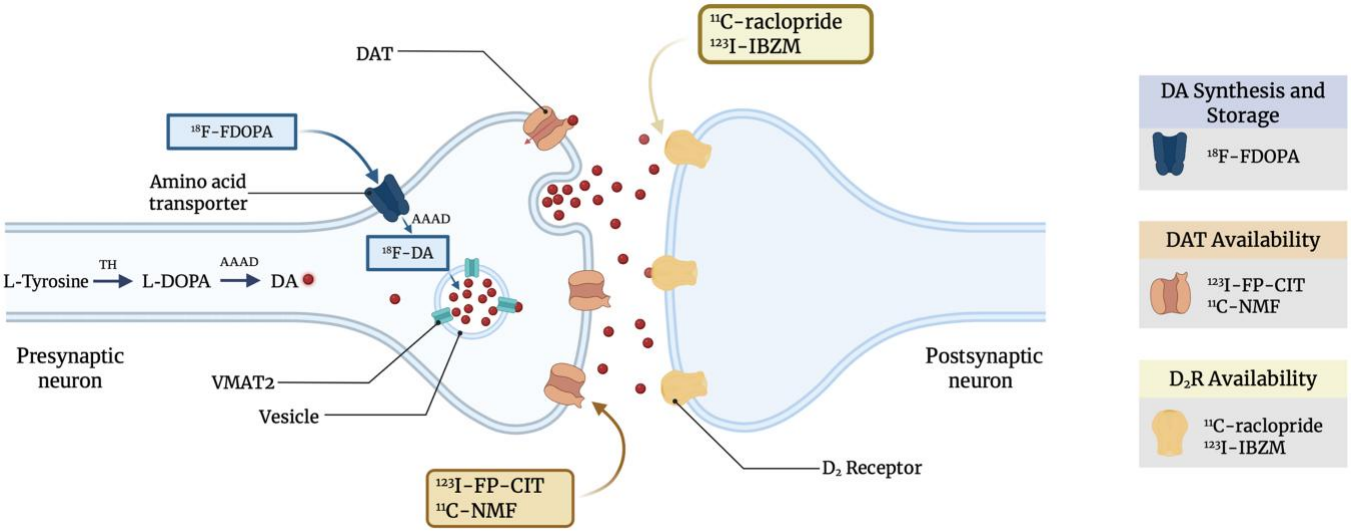
**Simplified representation of the dopamine synthetic pathway and radioligand action sites in a striatal dopaminergic synapse**. In the final two steps of the dopamine (DA) synthetic pathway, tyrosine hydroxylase (TH) converts tyrosine to L-DOPA, which is subsequently converted into DA by aromatic L-amino acid decarboxylase (AAAD). DA is stored in vesicles for tonic and phasic release. The diffusion of DA across the synaptic cleft and its binding to postsynaptic DA D_2_ receptors initiates intracellular cascades in the postsynaptic neuron that enable signal transmission. Following release, dopamine transporter (DAT) facilitates the reuptake of DA into the presynaptic neuron, which is then transported back into the vesicle by vesicular monoamine transporter type 2 (VMAT2). Selected PET/SPECT radioligands and their site of action are depicted: ^18^F-FDOPA is taken up into the presynaptic dopaminergic neuron via an amino acid transporter, then decarboxylated by AAAD into fluorodopamine (^18^F-DA) and temporarily stored in vesicles. Thus, ^18^F-FDOPA uptake can be interpreted as an indication of presynaptic DA synthesis and storage capacity. ^11^C-NMF uptake and ^123^I-FP-CIT binding indicate the reuptake capacity of DAT; ^11^C-raclopride and ^123^I-IBZM binding indicate postsynaptic D_2_ receptor availability. Owing to their competition with endogenous DA for binding, these radioligands can additionally derive an estimate of DA release by comparing ligand binding before and after a dopamine-stimulating event. AAAD = aromatic L-amino acid decarboxylase; ^11^C-NMF = *S*-^11^C-nomifensine; DA = dopamine; L-DOPA =L-3,4-dihydroxyphenylalanine/levodopa; DAT = dopamine transporters; ^18^F-FDOPA = 6-^18^F-fluoro-L-3,4-dihydroxyphenylalanine; ^18^F-DA = fluorodopamine; ^123^I-FP-CIT = *N*-ω-fluoropropyl-2β-carbomethoxy-3β-(4-iodophenyl)tropane/^123^I-ioflupane; ^123^I-IBZM = iodine-123-iodobenzamide; TH = tyrosine hydroxylase; VMAT2 = vesicular monoamine transporter type 2. Created in BioRender. Leong I. (2025) https://BioRender.com/ng1udyq.

Functional neuroimaging provides substantially more anatomical specificity than measurements of dopamine metabolites in CSF. PET and single photon emission computed tomography (SPECT) enable the quantification of dopaminergic transmission in the brain, as illustrated in Fig. 2. In ^18^F-FDOPA PET, radiolabelled DOPA is administered to participants and is subsequently converted by aromatic L-amino acid decarboxylase (AADC) to ^18^F-dopamine, which is sequestered within synaptic vesicles. Modelling of this uptake allows the calculation of the influx rate constant, K_i_, that serves as a measure of presynaptic dopamine synthesis capacity.^105^

In Parkinson’s disease, there is a convergence of both postmortem and neuroimaging studies showing that most degeneration in the nigrostriatal pathway preferentially affects the more caudolateral regions of the putamen.^106^ Significant reductions in putaminal ^18^F-FDOPA uptake and putaminal ^11^C-nomifensine (^11^C-NMF) binding (representing nigrostriatal nerve terminal reuptake site density^107^) correlate with the severity and lateralization of bradykinesia and rigidity.^108-111^ Relative to healthy controls, putaminal ^123^I-FP-CIT uptake levels (which represent density of the dopamine transporter) in patients with Parkinson’s disease correlate with the mean speed and amplitude of finger tapping, while caudate uptake levels correlate with mean amplitude.^112^

In early Parkinson’s disease, initial upregulation of striatal postsynaptic D_2_R shown by ^11^C-raclopride-PET and ^123^I-IBZM-SPECT (both of which bind to dopamine D_2_/D_3_ receptors) has been interpreted as a compensatory mechanism for presynaptic dopaminergic nerve terminal loss, followed by downregulation approximately 4 years following motor symptom onset.^113^ Though it remains difficult to ascertain whether downregulation results from disease progression or medication,^114^ consistent observations of higher striatal D_2_R binding contralateral to predominant motor features suggest that the initial upregulation is a temporary compensatory response to dopamine depletion resulting from presynaptic degeneration in early Parkinson’s disease.^113^ This is in accord with the inverse correlation observed between ^18^F-FDOPA uptake and ^11^C-raclopride binding in levodopa-naïve patients with Parkinson’s disease.^115^

Various lines of converging evidence suggest that a dopaminergic deficit in the sensorimotor striatum is specific to bradykinesia, rather than explanatory for all features of Parkinson’s disease. An ^18^F-FDOPA study found that uptake in the caudate nucleus was negatively associated with measures of executive functioning.^116^ Caudate presynaptic dopaminergic nerve terminal and transporter dysfunction correlates with slower cognitive speed and impaired verbal fluency.^116,117^ Bradyphrenia appears to be correlated with ^123^I-FP-CIT uptake in the head of the caudate.^118^ This is consistent with neuropathological findings showing reduced D1 receptor density in the caudate correlating with cognitive impairment.^119^ Anhedonia appears to involve both caudate and putaminal regions of the dorsal striatum in terms of dopamine transporter (DAT) binding.^120^ Using ^11^C-RTI-32 PET, a marker of dopamine and noradrenaline transporter binding, Remy *et al*.^121^ found a relationship between depression in Parkinson’s disease and lower binding in the locus coeruleus and the ventral striatum. The pathophysiology of the parkinsonian tremor does not appear to be primarily due to a dopaminergic deficit, nor does it correlate well with bradykinesia and rigidity.^122-124^ Thus, cognitive symptoms, affective symptoms and even tremor do not appear to depend specifically on the sensorimotor striatum in Parkinson’s disease in the way that bradykinesia does.

In parkinsonism secondary to dopamine antagonists, decreased ^123^I-FP-CIT binding in the putamen (a measure of dopamine transporter availability^125^) is present in a proportion of individuals^126^ and predicts levodopa response and parkinsonian symptom progression,^127^ with reduced putaminal ^18^F-FDOPA uptake observed in cases of continued or deteriorating parkinsonism post-withdrawal.^128^ This suggests that parkinsonism secondary to dopamine antagonists sometimes occurs in an at-risk population with subclinical presynaptic nigrostriatal dysfunctions.^129^ In neuroleptic malignant syndrome, ^123^I-IBZM-SPECT (a measure of postsynaptic D_2_R density^130^) reveals near-absent basal ganglia D_2_R binding of the ligand in the acute phase,^131^ indicating dopamine antagonist occupancy of postsynaptic D_2_R as a mechanism for reduced dopaminergic transmission associated with motor impairments.

Studies on catatonia mainly concentrate on presynaptic function, with limited data concerning postsynaptic D_2_Rs.^132^ Three ^18^F-FDOPA-PET studies of individuals with psychosis have each included a single patient with catatonia and have found decreased putaminal and caudate uptake in patients relative to other patients with schizophrenia and healthy controls,^133-135^ and comparable caudate K_i_ values to Parkinson’s disease,^136^ suggesting a similar presynaptic hypodopaminergia. Importantly, unlike D_2_R ligands, ^18^F-FDOPA does not appear to be modulated by D_2_R antagonists.^137^ However, it is not clear whether such observations represent a transient state or a longer-term trait. One small study of five patients suggested that dopaminergic abnormalities—at least presynaptically—are transient: five older women with catatonia and depression underwent a DAT-SPECT, which uses ^123^I-FP-CIT-SPECT to ascertain the density of DAT in the presynaptic terminal, finding reduced binding in the striatum, which improved after treatment with benzodiazepines or electroconvulsive therapy (ECT).^138^ One possible interpretation is that the reduction in DAT might be a compensatory response to reduced synaptic dopamine levels.

In depression, patients with motor retardation show lower left caudate ^18^F-FDOPA uptake,^139^ suggesting impaired presynaptic dopamine synthesis and storage. Studies investigating alterations in striatal DAT binding in depression have obtained mixed results.^140,141^ It should be noted that these studies did not specifically examine the relationship between DAT binding and PMR, and that the inconsistent findings may be due to the heterogeneity of depressed subjects included and medication effects. Studies of postsynaptic dopaminergic function in patients with depression and PMR have consistently found an increased binding of ligands to D_2_Rs compared to patients with depression without PMR, with studies examining striatal ^123^I-IBZM binding^142^ and putaminal ^11^C-raclopride binding.^143^ Concordantly, increased right striatal ^123^I-IBZM binding correlates positively with reaction or movement time and negatively with verbal fluency.^144^ Given that presynaptic dopamine production is impaired, this may either represent increased availability of D_2_Rs due to reduced levels of the endogenous ligand, or some compensatory response to a presynaptic deficit.

Overall, neuroimaging evidence supports the hypothesis that reduced dorsal striatal dopaminergic transmission is associated with psychomotor deficits across conditions. A summary of studies is included in Table 3, while Fig. 1B and D provides a visual comparison of studies in Parkinson’s disease and depression. In Parkinson’s disease, there is evidence that this is mainly related to the sensorimotor striatum (predominantly the putamen), but there is insufficient evidence from other conditions to localize the deficit so precisely. Postsynaptic alterations in depression, consisting of increased binding to D_2_Rs, parallel the findings in early-stage Parkinson’s rather than those in a later stage of the disease. Data on D_1_R binding are sparse, but are perhaps less relevant, given that available PET studies examining Parkinson’s disease patients typically observe no differences in striatal D_1_R binding compared to healthy controls.^145^ For Parkinson’s disease and PMR in depression, there is evidence implicating the caudate and putamen, but the role of the ventral striatum has been less well characterized. Evidence for drug-induced parkinsonism, neuroleptic malignant syndrome, catatonia, obsessional slowness and hyperkinetic disorders is less comprehensive and requires further research.

**Table 3:** Summary of imaging findings by synaptic region

Condition	Presynaptic findings		Postsynaptic findings	
	Tracer	Interpretation	Tracer	Interpretation
Parkinson’s disease	↓ putaminal ^18^F-FDOPA uptake^1-4^ ↓ putaminal ^11^C-NMF binding^1,3^	Reflects reduced presynaptic DA synthesis, storage and reuptake dysfunction	Initial ↑ ^123^I-IBZM and ^11^C-raclopride binding in striatum contralateral to predominant motor symptoms; subsequent ↓ binding 4 years after onset^5^	Initial ↑ D_2_R density may be compensatory mechanism for presynaptic DA nerve terminal loss. Later ↓ density may reflect chronic under-stimulation.
	Positive correlation between putaminal ^18^F-FP-CIT uptake and motor speed.^6^	Suggests reduced DA reuptake		
Drug-induced parkinsonism	↓ putaminal ^123^I-FP-CIT binding^7^ ↓ putaminal ^18^F-FDOPA uptake^8^	Suggests intrinsic ↓ in DA synthesis, which may imply that drug-induced parkinsonism tends to occur in an at-risk population with subclinical presynaptic nigrostriatal dysfunctions in DA storage and reuptake^9^	↓ putaminal ^11^C-raclopride binding^10^	↓ D_2_R binding, likely reflecting receptor occupancy by medications.
Neuroleptic malignant syndrome	No studies to the authors’ knowledge	No studies to the authors’ knowledge	Near absent basal ganglia ^123^I-IBZM binding in acute phase → near-normal binding despite persistent mild parkinsonian symptoms after 4 months^11^	Suggests high postsynaptic D_2_R occupancy is a mechanism for akinesia
Catatonia	↓ putaminal^12,13^ and caudate^14^ ^18^F-FDOPA uptake Initial ↓ ^123^I-FP-CIT binding → Restored binding post- ECT/benzodiazepine treatment^15^	↓ DA synthesis and storage. Reversible ↓ in DAT capacity.	No studies to the authors’ knowledge	No studies to the authors’ knowledge
Psychomotor retardation in depression	↓ left caudate ^18^F-FDOPA uptake in PMR patients relative to HCs and non-PMR patients^16^	↓ DA synthesis and storage in left caudate	Initial ↑ striatal ^123^I-IBZM binding pretreatment compared to HCs and non-PMR patients; subsequent ↓ post-treatment in PMR patients relative to healthy controls and non-PMR patients^17^ ↑ putaminal ^11^C-raclopride binding compared to HCs^18^	Suggests either ↑ availability of D_2_R due to ↓ synaptic DA levels, or a compensatory ↑ in D_2_R density. Treatment may result in normalization.

^11^C-NMF = ^11^C-nomifensine; ^18^F-FDOPA = ^18^F-dihydroxyphenylalanine; ^123^I-FP-CIT = ^123^I-*N*-3-fluoropropyl-2β-carbomethoxy-3β-(4-iodophenyl)nortropane; ^123^I-IBZM-SPECT = ^123^I-iodobenzamide single photon emission computed tomography; DA = dopamine; DAT = dopamine transporter; D_2_R = dopamine D_2_ receptor; ECT = electroconvulsive therapy; HC = healthy control; PMR = psychomotor retardation.

## Dopaminergic treatments

L-DOPA, the immediate precursor of dopamine, is the mainstay of treatment for Parkinson’s disease, acting through supplementing deficient dopamine synthesis.^146^ Other effective pharmacotherapies include dopamine agonists and monoamine oxidase inhibitors, which inhibit dopamine catabolism.^147-152^ Observational data suggest that dopamine agonists improve motivational symptoms while monoamine oxidase inhibitors improve both depressive and motivational symptoms in Parkinson’s.^153^ A large randomized controlled trial of the dopamine agonist pramipexole versus placebo in Parkinson’s disease found an improvement in depression that was largely not mediated by improved motor symptoms.^154^

Conversely, antipsychotics that act as antagonists at the dopamine D_2_R can induce parkinsonism, with typical antipsychotics posing greater risk.^21^ Antipsychotic-induced parkinsonism can be treated with the dopamine agonist rotigotine or amantadine, which enhance dopamine synthesis and release.^155-157^

Neuroleptic malignant syndrome is an adverse effect of antipsychotic treatment, with typical antipsychotics and higher doses associated with increased risk.^20,158^ There is evidence that the dopamine agonist bromocriptine can be used to treat neuroleptic malignant syndrome,^159,160^ and a case series suggested levodopa may also be beneficial.^161^ Discontinuation of pro-dopaminergic medication in patients with Parkinson’s disease has been shown to cause neuroleptic malignant syndrome-like symptoms (parkinsonism-hyperpyrexia syndrome).^162-166^

The use of antipsychotics in catatonia is controversial because of a frequent perceived need to treat underlying psychosis and reported benefits in such circumstances.^167^ Aside from the risk of inducing neuroleptic malignant syndrome,^86^ antipsychotics can induce or worsen catatonia, with typical antipsychotics posing a greater risk, postulated to be due to their higher affinity for D_2_R.^87^ Antipsychotics can simultaneously induce catatonia and parkinsonism.^168^ Clozapine, which has a unique cholinergic mechanism and weak D_2_R affinity, may be an exception that is effective in catatonia,^169,170^ with clozapine withdrawal sometimes precipitating catatonia.^171^ Conversely, initial evidence from one small human study and two animal models, suggests levodopa may be beneficial for treating catatonia, although worsening psychosis has limited its use.^172-174^ Various case reports also support the use of amantadine and methylphenidate.^175-178^

In depression, there is evidence for benefit in mood from several randomized controlled trials for the dopamine agonist pramipexole,^179,180^ though studies have not investigated PMR as an outcome. There is less evidence that levodopa and amineptine (a dopamine releasing agent and reuptake inhibitor^181^) are effective at alleviating PMR in depression.^22,182-184^ Bupropion, a noradrenergic and dopamine reuptake inhibitor, has evidence from numerous clinical trials supporting its superiority to placebo in depression.^185^ Interestingly, poor mental processing speed in depression has been shown to be a predictor of strong response to the bupropion.^186,187^ Aripiprazole, a dopamine partial agonist, has evidence for augmentation of antidepressant response in treatment-resistant depression, although studies have not examined PMR specifically.^188^ Inhibitors of monoamine oxidase A, which reduce reuptake of a range of neurotransmitters including dopamine, are also effective in depression, though little is known about predictors of response.^189^

Another treatment predominantly used for depression that merits a mention is ECT, the use of an electrical stimulus to induce a generalized seizure.^190^ ECT is associated with in volumetric increases in the nucleus accumbens and caudate nucleus, correlating with improved PMR in depression.^191,192^ Similarly, a relative increase in metabolism in basal ganglia regions have been detected following ECT.^193^ A mean reduction in striatal DAT binding of 13.1% and reduced dopamine autoreceptor sensitivity following ECT are observed.^194,195^ Interestingly, ECT in Parkinson’s disease transiently improves motor function, even when patients with prior psychiatric morbidity are excluded.^196,197^ However, the mechanisms of ECT remain poorly understood, likely involving multiple neurotransmitters.^198,199^

Overall, dopamine antagonists tend to induce and worsen PMR, while it is ameliorated by medications that enhance dopamine transmission, either by increasing dopamine release or inhibiting its breakdown. However, the evidence in catatonia and depression remains preliminary with small sample sizes and a lack of well-conducted clinical trials. A summary of the impact of dopaminergic medications on different neuropsychiatric conditions is shown in Table 4. While it is plausible that ECT acts through dopaminergic mechanisms, there remains a great deal of uncertainty.

**Table 4:** Comparison of diseases characterized by psychomotor retardation and response to dopaminergic medications

Condition	Levodopa	Dopamine agonists	Dopamine antagonists (antipsychotics)	Other
Parkinson’s disease	Effective^192^	Effective^151-153^	Deleterious^193,194^	Monoamine oxidase inhibitors are effective^154,156^
Neuroleptic malignant syndrome	Effective^165,a^	Bromocriptine is effective^163,164^	Causative^161^	—
Drug-induced parkinsonism	Ineffective^195-197^	Rotigotine is effective^159^	Causative^16^	Amantadine is effective^158^
Catatonia	Effective^175-177,a^	Ineffective^198,a^	Controversial^25^; clozapine may be effective^172^	Methylphenidate is effective^178-180,a^
Depression with psychomotor retardation	Effective^184-186,a^	Effective^157,182,a^	Aripiprazole and quetiapine are effective^190,199,200^	Bupropion is effective^188^

^a^Based on limited evidence.

## Cognitive neuroscience

We have reviewed the evidence that striatal dopamine transmission plays a transdiagnostic role in PMR, beyond the more established case of Parkinson’s disease. The question remains as to whether this is plausible given what we understand about the neurobiology of dopamine transmission in the striatum.

Dopamine has a critical role in self-initiated movement across various brain regions.^200^ Rather than directly controlling fine movement kinematics, its influence lies in the initiation and execution of deliberate actions.^201^ There are transient increases in nigrostriatal dopaminergic neuronal activity that consistently precede movement initiation or acceleration in mice, which correlate with the vigour of future movements.^202^ Mesolimbic dopaminergic transmission is also key to causal learning through retrospective associations^203^ and prediction error calculations.^204^ The ventral tegmental area’s potential downstream effects on the ventral striatum have been increasingly recognized in Parkinson’s disease, particularly for non-motor symptoms.^205^ Considerable evidence indicates the nucleus accumbens as a ‘limbic-motor interface’ that translates motivation into action.^206,207^

However, it is the dorsal striatum that is thought to be central in ‘action-energizing’ movement. Tonic dopamine levels in the dorsal striatum encode motivational signals that reflect the energetic cost of movement (i.e. the appropriate allocation of energy to match the demands of the task).^208^ Despite similar movement parameters (i.e. trajectory quality, end point accuracy, energy expenditure) to healthy controls, patients with Parkinson’s disease exhibit slower movement with increased energetic demands,^208^ leading to bradykinesia being conceptualized as ‘implicit’ motor motivation deficits.^209^ In other words, the slow movement observed in Parkinson’s disease is due to a lack of motor vigour, rather than an inability to move quickly or a simple speed-accuracy trade-off. Patients prefer slower movements because they are more sensitive to the energetic cost of faster, more demanding movements.^210^ Similar impairments in depression and schizophrenia suggest that movement speed reflects implicit evaluations of movement energy costs signalled by striatal dopamine.^211^ Additionally, reduced dorsal striatal DAT binding, indicative of presynaptic neuronal death, is related to anhedonia and apathy.^120,212^ Considered collectively, motor speed may reflect a paucity of motivational drive.

These findings could reflect how striatal dopaminergic dysfunction affects effort-related decision-making by impairing the evaluation of motor energy costs. In Parkinson’s disease, the loss of dorsal striatal dopamine function weakens the connection between motivation and movement gain.^213^ This is consistent with the striatal dopaminergic system’s role in reward-based learning, where dopaminergic neurons encode a discrepancy between expected and obtained rewards of an action.^214^ This ‘reward prediction error’ (RPE) signal effectively guides learning until the reward gain is maximized.^215^ Though these processes have typically been associated with the ventral striatum,^216^ recent evidence implicates the involvement of the dorsal striatum, with the putamen and caudate nucleus potentially implicated in different roles.^217-219^ A gradient in the speed of reuptake of dopamine has been reported from the ventral to dorsal components of the striatum, with the dorsal striatum more sensitive to the temporally precise phasic signals and encoding prediction errors than the ventral striatum.^220,221^ The putamen appears to be important for the encoding and learning of stimulus-action associations,^222^ while the caudate nucleus is involved in the encoding of RPEs during goal-directed behaviour.^223^ These regions work in parallel to integrate sensorimotor, cognitive and motivational emotion that support action selection and initiation in goal-directed action.^224-226^ Thus, striatal dopamine release signals the value of actions, facilitating both motor learning and the invigoration of actions associated with motivationally significant outcomes, all of which are very relevant to psychiatric conditions such as depression and catatonia.

Beyond effort evaluation, decision-making about movement also involves deliberation, during which the individual evaluates different courses of action over time. Deliberation involves processes such as working memory, rule extraction and outcome prediction. Based on this definition, Pessiglione *et al*.^227^ noted a disrupted temporal uncoupling between mental deliberation and motor execution in unmedicated Parkinson’s disease patients relative to healthy controls, which was reversed by levodopa but not by subthalamic nucleus deep brain stimulation. However, as this study was not accompanied by molecular imaging, the precise site and mechanisms of dopamine depletion remain unclear. The findings also led to speculation that the loss of functional segregation between basal ganglia circuits may explain the loss of temporal uncoupling between deliberation and execution, leading to decision-related hesitations being expressed as movements.

Notably, the striatal subregions may perform different functions depending on the types of stimulus inputs (e.g. conditioned versus unconditioned, external versus internal), and the parameters of motor control (e.g. large- versus small-scale, locomotion versus manipulation) being governed.^228^ Thus external sensory stimuli can be effective in facilitating gait initiation in Parkinson’s patients with freezing of gait,^229-231^ particularly visual cues in improving gait speed.^232,233^ Interestingly, it has been shown that akinetic Parkinson’s patients can show a sudden temporary ability to move in situations of intense emergency, so-called ‘kinesia paradoxa’.^234^ These findings suggest that difficulties in movement initiation could be overcome by altering the sensory input, and that the fundamental impairment in Parkinson’s disease may be rooted in the execution of actions in response to the individual’s intentions.^235^ Catatonic signs can similarly fluctuate within an illness episode.^236,237^

There is also a reduced capacity to switch between different motor programs within a broader motor plan in Parkinson’s disease.^238^ Specifically, impairments in sequential and simultaneous movements are more pronounced compared to individual movements.^239^ Therefore, the inability to switch between motor plans may be a process underlying the reduced capacity to generate spontaneous voluntary action and the pronounced slowness in Parkinson’s disease.

Relatedly, the nigrostriatal network may be implicated in the initial learning of sequential motor tasks and the memory processes enabling recall of learned motor sequences. Specifically, striatal dopaminergic transmission may be involved in the acquisition of predictive movement sequences and the efficient predictive retrieval of succeeding movements to form complete goal-oriented action sequences in monkeys.^240^ Moreover, there is evidence of striatal dopamine release during performance of a motor sequential learning task in humans. Measurements of dopamine D_2_R availability by ^11^C-raclopride have revealed that activation in the left putamen and right caudate is associated with response selection and execution, while activation in the left caudate is associated with detecting contextual changes.^241,242^ Taken together, these findings may be interpreted as a plausible explanation for the difficulties in motor plan switching experienced by patients with Parkinson’s disease, and also aligns with the suggestion that striatal processing enables the ‘chunking’ of sequential acts into performance units.^243^

In summary, it is possible that dysfunction of dopaminergic transmission in the dorsal striatum (with stronger motor correlates) more directly affects movement parameters such as frequency, speed and control. In contrast, dysfunction in the ventral striatum (with stronger RPE correlates) may influence movement indirectly by affecting cognitive functions like motivation, decision-making and learning. Therefore, it may be that the striatal subregions operate hierarchically to contribute to PMR. This view may be supported by the temporal gradient in dopamine degeneration observed in Parkinson’s disease, with the dorsal striatum being affected in early stages of the disease, progressing to the ventral striatum in later stages of the disease.^244,245^ However, it would be an oversimplification to associate the dorsal striatum solely with motor functions and the ventral striatum strictly with limbic or motivational functions. Ventral striatal dopaminergic depletions still impact motor parameters by reducing the overall frequency and speed of various activities. Although further research is needed to clarify the roles of these striatal subregions, dopamine transmission remains a central mechanism underlying both motor and motivational control.

## Discussion

In this review, we have synthesized the evidence that reduced striatal dopamine transmission is a transdiagnostic substrate for PMR. The overlapping clinical features across conditions imply striatal dopamine as a common substrate for PMR. Epidemiological evidence indicates that transdiagnostic PMR in clinical populations may result from predisposed dopaminergic dysfunction or antidopaminergic medication effects. Molecular neuroimaging and laboratory evidence mainly associate reduced dorsal striatal transmission with PMR. Dopaminergic medication appears effective in various conditions with PMR.

Mesolimbic dopaminergic transmission is key to causal learning through retrospective associations,^203^ and prediction error calculations.^204^ The ventral tegmental area’s potential downstream effects on the ventral striatum are increasingly recognized in Parkinson’s disease, particularly in relation to cognitive and affective symptoms.^205^ Considerable evidence indicates the nucleus accumbens as a ‘limbic-motor interface’ that translates motivation into action.^206,207^ However, dorsal striatal dopaminergic activity remains central for ‘action-energizing’ movement. Motor speed may reflect a reduction in motivational drive due to reduced striatal dopamine signalling.

Although this review has focused on dopamine, it would be an oversimplification to attribute the full spectrum of motor features of a diverse range of conditions to a single neurotransmitter. In Parkinson’s disease, while it is accepted that non-dopaminergic mechanisms are central to non-motor symptoms,^246^ it is also clear that other neurotransmitters are involved in motor symptoms, although their role is probably less straightforward than that of dopamine. PET studies have found that serotonergic terminals are depleted early in the course of Parkinson’s disease, supporting findings of older post-mortem studies,^247^ but serotonin transporter availability does not seem to correlate with motor symptoms.^248^ While the serotonin receptor agonist *meta*-chlorophenylpiperazine (mCPP) results in reduced locomotor activity in rodents,^249,250^ in healthy humans this effect appears to be driven primarily by cognitive slowing rather than a direct motor effect.^251^ Cholinergic dysfunction has also been implicated in the motor symptoms of Parkinson’s disease, but this is primarily in posture and freezing of gait, rather than in bradykinesia.^252^ Cortical GABA levels—as measured in magnetic resonance spectroscopy—do not appear to differ between individuals with Parkinson’s disease and healthy controls, but one study did find an inverse correlation between GABA in the motor cortex and motor symptoms.^253^

In catatonia, there is evidence both from the effectiveness of benzodiazepines^254^ and from SPECT imaging showing a reduced density of GABA-A receptors in the left sensorimotor cortex^255^ that GABA is also central to the pathophysiology. It is increasingly recognized that there are collections of neurons in the human CNS that can co-release both GABA and dopamine.^256^ In depression, it is likely that a range of monoamines are relevant^257^ and a reciprocal relationship between serotonin and dopamine release in the substantia nigra is observed.^258^ In ECT, glutamate and GABA release mediated through serotonin receptor sensitization modulates dopamine function.^259^ A pivotal role for dopamine therefore is not to the exclusion of other neurotransmitters, and there are clear relationships in the striatum between dopamine and transmitters including acetylcholine, glutamate and GABA. However, we focus on dopamine because this seems to be where the preponderance of evidence is and where there is clearest transdiagnostic relevance.

While PMR may be associated with dorsal striatal dopamine transmission downregulation, it is less clear—or less straightforward—that increased striatal dopamine transmission is linked to psychomotor agitation, as evidenced by neuroimaging findings and clinical observations of co-existing PMR and psychomotor agitation.^260^

One limitation to our findings is the heterogeneity of measurement of PMR in the evidence underpinning our review.^6^ Distinguishing cognitive from motor variables is challenging, as it introduces confounders such as executive functions, memory and attention.^7,261^ PMR scales, despite good psychometric properties, are susceptible to bias due to unnaturalistic settings.^7,38^ Actigraphy, measuring activity level over longer periods, provides a more naturalistic and objective alternative but may not correlate with other behaviours.^38^ Advances in instrumentation and measurement modalities used in conjunction may enhance ecological validity and identify underlying brain circuitry.^262^

One important implication from this review is that current diagnostic classifications of psychiatric disorders may be grouping together disorders that are highly heterogeneous in their pathophysiology. Depression may or may not feature PMR. Catatonia may present with a stuporous-retarded phenotype, but it may equally in current classifications present with an excited-agitated phenotype.^2,44,263^ We propose that future studies of psychiatric disorders with prominent motor phenotypes consider restricting their samples to a certain phenotype or stratifying the analysis by that phenotype. Given the heterogeneity of depression and low response rates to first-line therapy, the need for new and effective therapeutic interventions cannot be understated. Further studies are required to determine if PMR predicts response to dopaminergic antidepressants (e.g. bupropion and monoamine oxidase inhibitors). Although initial evidence suggests levodopa and dopamine agonists alleviate PMR in depression, recent larger studies have been retracted due to methodological issues.^264,265^ Based on our research, we propose reattempting these studies and conducting larger trials with dopamine agonists in selected populations by motor phenotype as a way of avoiding grouping patients with divergent pathophysiology. This approach may also benefit akinetic catatonia, as some evidence indicates the efficacy of levodopa and methylphenidate.^172-174,266^ ECT is recommended for patients with PMR, and/or resistant to pharmacological treatment, but is underused due to stigma.^267-269^ Further studies about its mechanism could dispel misconceptions, and dopamine transmission is a promising candidate.

Another implication concerns the imaging modalities we should consider employing in research on neuropsychiatric disorders. In Parkinson’s disease, we can localize bradykinesia quite precisely to the dorsolateral striatum, although in other conditions, there is less certainty and anatomical specificity. Considering the difficulty of obtaining consent for invasive procedures and having severely unwell patients remain still in a scanner for prolonged periods, future research could explore alternative imaging methods. One option may be ^18^F-(*E*)-*N*-(3-iodoprop-2-enyl)-2β-carbofluoroethoxy-3β-(4′-methyl-phenyl) nortropane [(^18^F-FE-PE2I)-PET], which shows comparable sensitivity and specificity with superior striatal DAT specificity to ^123^I-FP-CIT-SPECT.^270,271^ Its faster kinetics enable shorter intervals between injection and imaging (^18^F-FE-PE2I: ≤ 20 min, ^123^I-FP-CIT: ∼30 min) and shorter static imaging protocols (16.5–42 min).^270,272^ There is also a fast ^18^F-DOPA PET imaging protocol,^273^ which may be better tolerated by unwell patients. Another option is neuromelanin MRI (NM-MRI), increasingly validated as a non-invasive, cost-effective measure of presynaptic dopaminergic neuron density and function, although it is less able to capture transient states.^274^ NM accumulates in the midbrain nuclei throughout the lifespan,^275^ reflecting a more stable marker of dopamine synthesis compared to the state-specific data from PET/SPECT.^276^ NM-MRI signals correlate with PET/SPECT measures of striatal dopamine terminal loss,^277-279^ dorsal striatal dopamine release^280^ and putaminal dopamine synthesis in Parkinson’s disease,^281^ showing decreased NM signal intensity in the substantia nigra pars compacta compared to healthy controls.^282-285^ However, its applicability in other conditions requires further research. If reduced striatal dopamine transmission accounts for PMR across conditions, similar decreases in NM signal intensity and striatal DAT binding would be expected in catatonia and some forms of depression if performed when the patient is unwell. It is not clear whether the pathology in the substantia nigra in Parkinson’s disease is mirrored in other conditions featuring psychomotor retardation, which may entail functional rather than structural deficits.

The evidence examined in this narrative review generally supports reduced dorsal striatal dopamine transmission as a transdiagnostic substrate for PMR across various neurological and psychiatric conditions. That is, reduced dopamine transmission could contribute to the pathophysiology of PMR. While current evidence is insufficient to conclusively define anatomical and mechanistic specificities, we suggest that presynaptic dysfunction in dopamine storage and reuptake in the dorsal striatum affects downstream neurotransmission, resulting in PMR. However, while the role of dopamine in Parkinson’s disease is compelling, there are substantial gaps in the literature for psychiatric conditions, which could be filled by further neuroimaging and a clinical trial of a dopaminergic medication in patients with depression and PMR.

Striatal dopamine should be considered alongside other interacting neurotransmitters, with further investigation needed into medication action sites and the anatomical and mechanistic specifics of striatal transmission. Future research should move beyond static models to explore striatal dopamine's role in PMR as part of a dynamic, complex system with nuanced roles of striatal subregions and the interplay with other neurotransmitter systems.

## Supplementary Material

awaf335_Supplementary_Data
