# Supplementary material for: Reduced striatal dopamine transmission as a transdiagnostic substrate of psychomotor retardation
Source: Brain. 2025 Sep 12;149(1):18–36. doi: 10.1093/brain/awaf335 (PMC12782176; doi:10.1093/brain/awaf335)
Supplement: awaf335_Supplementary_Data [file awaf335_supplementary_data.pdf]

Author Contributions

JPR conceptualized the review and led the writing of the original draft. ILL, KS and THN reviewed the literature and wrote a first draft of the manuscript. All authors contributed to the development of the manuscript and have read and approved the final version of the manuscript.
